# Coincidence between Transcriptome Analyses on Different Microarray Platforms Using a Parametric Framework

**DOI:** 10.1371/journal.pone.0003555

**Published:** 2008-10-29

**Authors:** Tomokazu Konishi, Fumikazu Konishi, Shigeru Takasaki, Kohei Inoue, Koji Nakayama, Akihiko Konagaya

**Affiliations:** 1 Department of Bioresource Sciences, Akita Prefectural University, Shimosinjyo Nakano, Akita, Japan; 2 Genomic Sciences Center, RIKEN, Tsurumi-ku, Yokohama, Kanagawa, Japan; 3 Research Division for Advanced Technology, Mitsubishi Chemical Safety Inst. Ltd., Kamisu, Ibaraki, Japan; 4 Department of Computer Science, Tokyo Institute of Technology, Meguro-ku, Tokyo, Japan; University of Calgary, Canada

## Abstract

A parametric framework for the analysis of transcriptome data is demonstrated to yield coincident results when applied to data acquired using two different microarray platforms. Microarrays are widely employed to acquire transcriptome information, and several platforms of chips are currently in use. However, discrepancies among studies are frequently reported, particularly among those performed using different platforms, casting doubt on the reliability of collected data. The inconsistency among observations can be largely attributed to differences among the analytical frameworks employed for data analysis. The existing frameworks are based on different philosophies and yield different results, but all involve normalization against a standard determined from the data to be analyzed. In the present study, a parametric framework based on a strict model for normalization is applied to data acquired using several slide-glass-type chips and GeneChip. The model is based on a common statistical characteristic of microarray data, and each set of chip data is normalized on the basis of a linear relationship with this model. In the proposed framework, the expressional changes observed and genes selected are coincident between platforms, achieving superior universality of data compared to other frameworks.

## Introduction

The transcriptome, the contents of mRNA, determines the functions of a cell. Microarrays are currently widely used to acquire comprehensive transcriptome information, and thus have greatly facilitated transcriptome research. However, an appropriate intellectual framework for systematizing the data collected using various microarrays [1] has yet to be developed. An intellectual framework is a set of basic assumptions or fundamental principles [2] that structures the evaluation process. Many measurements in the natural sciences conform to a framework based on a universal system of rules, such as the International System of Units. For the many different types of microarray platforms, however, it is difficult to transform raw data so as to accord with any one of the existing frameworks, precluding reliable comparisons among dissimilar data sets. Consequently, some form of data normalization is required for processing microarray data in order to allow intercomparison of raw data. Data normalization is generally performed by finding certain definable characteristics in the data that with appropriate calculations could be used to unify dissimilar data sets. The characteristics to be unified, the standards, and the set of calculations are prescribed by an intellectual framework, the basis of which is the data normalization scheme. Many normalization methods have been proposed for microarray studies, each with a different set of basic assumptions. The dissimilarity of the normalization methods and assumptions constituting the intellectual frameworks thus result in discrepancies when comparing measurements obtained using different frameworks. As there presently exists no framework that yields consistent results among different platforms, the reliability of numerous measurements in the literature may have been compromised, particularly when comparisons among different platforms have been performed, which has raised many questions and criticisms [3]–[7].

Developing a universal framework for microarray analyses has proved to be more problematic than may have been expected. The essential character of a transcript is determined by its concentration, as the transcript acts as a template for the translation process, and the rate of translation is linear when compared to the concentration of the template in the cytosol. However, concentrations cannot be measured using present microarray systems. Measurement of transcripts requires that RNA samples be isolated from tissue, for which the collection rates and cytosol volumes are difficult to estimate. Consequently, even if the mass of each transcript in a sample can be determined, the concentrations cannot be calculated. This practical imprecision is further complicated by the variety of platforms available for microarray systems, which differ with respect to the probe sensitivity of the hybridization systems and the nucleotide sequences employed. The potential errors and biases will also differ between platforms, and the level of additive noise and saturation will vary according to the measurement approach. Such noise and error contribute to further discrepancies among data sets. To achieve universality of data and resolve the problems associated with incompatibility, a unified intellectual framework is therefore required. Without an adequate framework that is not affected by measurement sensitivity and background, even the ratios of expression levels cannot be estimated correctly as these are framework dependent. However, relatively little attention has been paid to the development of such a framework in microarray analyses [1].

A universal intellectual framework for microarray data analysis should have the capacity to compensate for differences in measurement related to the differences among platforms and/or wet procedures. A parametric framework is expected to be suitable for achieving such universality of data by allowing the transcriptome to be described in terms of parsimonious models based on thermodynamic models for the formation of the transcriptome in a cell [8] and the detection of RNA by hybridization [1]. In such a framework, data could be normalized with respect to a statistical characteristic common to all measurements. The lognormal pattern of the data distribution [9] has been proposed as a promising basis for a parametric framework. By this approach, an appropriate background specific to each hybridization is first subtracted from the data. The logarithms of the background-corrected data are then modified by subtracting a parameter representing the center of the distribution (μ), and then by dividing by a parameter representing the width of the distribution (σ). The normalized data sets are then compared to a lognormal distribution model to verify the suitability of the applied model. This approach allows the normalized data to be compared with other normalized data on the basis of linear characteristics. Measurements can also be made to test the lowest value unaffected by additive noise and the highest value unaffected by saturation, since the data diverge from the expected pattern at these limits [9]. The differences among normalized data can then be evaluated using generalized linear models [1], [8], [9].

In the present study, the universality of a parametric framework is tested by comparing data acquired using several different microarray platforms. While differences in the measurement positions of each transcript could alter the obtained information, the overall trend in the information obtained from platforms should coincide. To test the coincidence, a series of data were obtained from a rat toxicology project study [10]. Multi-sample RNA isolated from rat organs were then hybridized to two platforms: an in-house microarray (ToxArray III), and the GeneChip microarray in three different laboratories [11], [12]. The ToxArray III is a typical microarray on slide glass, consisting of a single 60 mer probe per gene, and two samples are measured simultaneously per chip. In contrast, the GeneChip consists of 11 perfect match (PM) and miss match (MM) 25 mer probes per gene, and a single sample is measured per chip. In this report, the coincidence of information is checked by examining the measured logarithmic ratios and gene candidates that may be affected by Safrol [12] treatment. An additional series of data was obtained from an inter-platform comparison study [13] in which two pooled mouse RNA samples were hybridized to various slide-glass and GeneChip platforms. All data were normalized using a parametric framework based on a three-parameter lognormal distribution model [1], [9]. In order to evaluate the methodology, GeneChip data were also normalized using both MAS5 [14] and RMA [15], slide-glass arrays with a two-color system were normalized by the LOEWSS method [16], and slide-glass arrays with a single-color system were normalized by quantile normalization [15]. MAS5 is the original method described by the manufacturer and involves classification of genes into “Present”, “Marginal”, and “Absent” in addition to normalization and summarization of data, and is a complex framework consisting of many conditional branches. RMA is a widely used alternative based on the quantile method [15], in which data distributions of subject data sets are unified by replacing the entire data set with average data while maintaining the orders in each data set. LOWESS [16] cancels correlative trends between signal intensities and logratios by dividing each pair of data with customized functions. Although these frameworks are widely used in transcriptome studies, all have a critical drawback in that the appropriateness of the assumptions comprising the frameworks have not been verified or are intrinsically difficult to verify. Most of the assumptions introduce for the conditional branches in MAS5 lack experimental evidence, and one of the most basic assumptions, that the amount of transcript is linear to the PM-MM value, has been proved to be erroneous [1]. Both quantile normalization and LOWESS invariably introduce some desired character or bias to the data, making it difficult to develop appropriate calculations for verification of the assumptions.

In the test of frameworks, superior coincidence is demonstrated using the parametric framework. The proposed framework thus appears to provide a means for the seamless integration of information obtained in transcriptome studies. The highly reliable data thus obtained may also provide clues for decoding the hereditary traits within the genome [8], which may in turn lead to rapid progress in the life sciences.

## Results

### Data distribution

**Figure 1:**
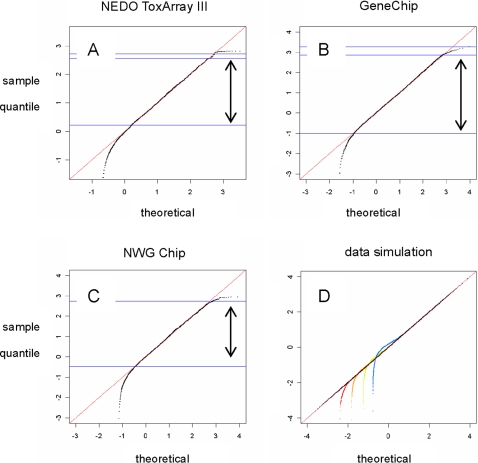
QQ plots showing the distribution of normalized data for signals obtained for the same RNA sample. (A) NEDO ToxArray III spots (without controls), (B) GeneChip PM data from the toxicology study, and (C) MWG chip data from platform comparison studies. Red line denotes *y* = *x*, and arrows denote valid range of data with respect to model fit. (D) Simulated data distribution. Lognormally distributed signals form a straight line on the QQ plot (black), while those with various levels of normally distributed noise fall in the lowest range (colored dots). Interactive commands used in the *R* simulation are provided in Supporting Information S1.

The statistical characteristics of the data were determined using conventional quantile-quantile (QQ) plots (Figure 1 and Supporting Information Figure S1). The QQ plot shows the degree of coincidence between the data distributions of two numeric groups. The distribution of logarithms of microarray data (*y* axis) is compared in Figures 1 and S1 with the theoretical normal distribution (*x* axis), that is, sorted normalized data (sorted *z* scores) are plotted against the theoretical values. The *y* = *x* line thus represents complete coincidence between the data distribution and the theoretical prediction, while additive noise in the raw data will bend the linear relationship in the weakest intensity range. Although the signal intensity follows the theoretical distribution pattern over a certain range for both chips, there is a marked difference in the valid intensity range between the two chips. The narrower valid range for ToxArray data suggests a higher level of additive noise. The distribution of ToxArray data has a larger scale parameter (σ) than that of GeneChip data, with median values of 1.02 and 0.685, respectively. The dynamic range of signals, estimated from the ratio of the strongest to weakest signal for 10 000 measurements, is 2×10^5^ for GeneChip, and 1×10^8^ for ToxArray. The higher σ value is likely to result in measurements exceeding the limits of the scanner, which usually covers a range of 10^4^–10^5^. Additionally, unevenness in hybridization, as observed from the pseudo images [17], was substantially higher for ToxArray (see Supporting Information Figure S2).

### Coincidence of logarithmic ratios between chip platforms

**Figure 2:**
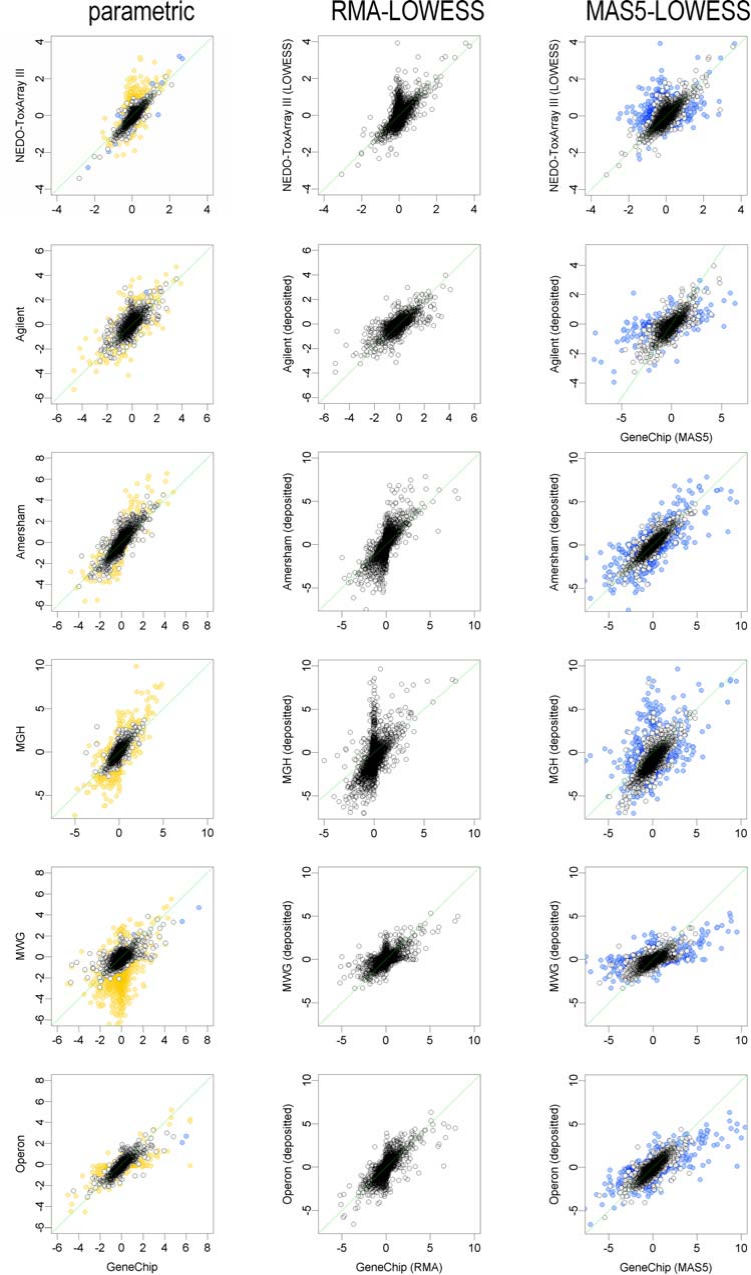
Coincidence in log_2_ ratio among estimations of expressional differences for slide-glass-type chips and GeneChip. Data plotting on the *y* = *x* line (green) are coincident between platforms. Signals out of the valid range of the parametric and MAS5 framework are plotted in blue (GeneChip) or orange (slide-glass).

For the genes common to slide-glass-type chips and GeneChip, the logarithmic ratios determined by different frameworks are compared in Figure 2. In the parametric framework, the logarithmic ratios are coincident in the valid signal range (Figure 2, left). Outside of the valid range, however, data obtained using slide-glass-type chips become substantially divergent, while the GeneChip data remain relatively close to the *y* = *x* line. This may indicate the noise reduction effect associated with the GeneChip due to the averaging of multiple PM cells for each gene. In contrast, larger differences were observed between the LOWESS- and RMA-normalized data (Figure 2, center and right). The coincidence between LOWESS and MAS5 results is very poor for data labeled “Absent” in MAS5, but improved coincidence was observed for the “Present” data, although such a relationship should not always be expected; for example, almost no coincidence was observed in other cases (see Supporting Information Figure S3).

### Coincidence in selected genes

**Figure 3:**
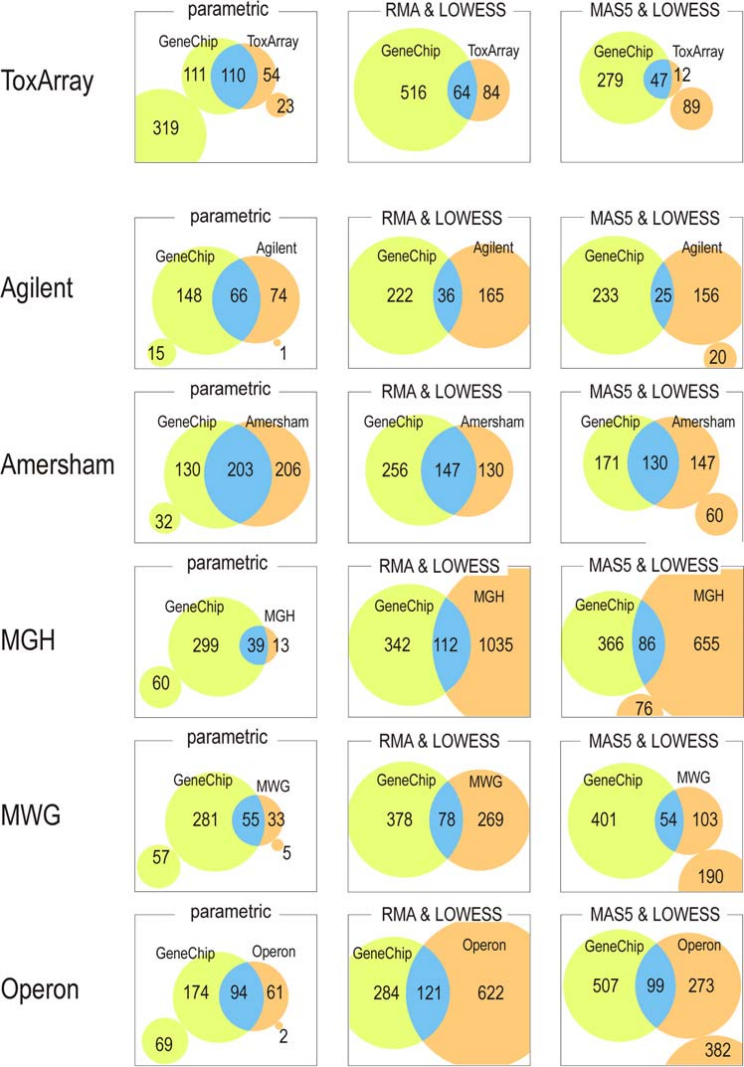
Summary of selected genes showing coincidence between platforms. Values denote number of genes selected for each of the array platforms. Signals outside of the valid model range are omitted (parametric and MAS5 framework). Isolated areas represent the genes that could not be determined in the other platform due to the limitation of signal range.

The lists of genes exhibiting expressional changes larger than the threshold levels determined by noise level estimations are compared in Figure 3. The numbers of selected genes correspond to the estimated magnitude of differences and fluctuations in measurements. Using the parametric framework, a larger overlap and smaller disagreement between the lists for each platform are obtained. This comparison also reveals differences in the detection power of the chips. For example, 319 genes selected by GeneChip were out of the detection range of ToxArray. Comparisons between LOWESS and RMA or MAS5 methods resulted in a markedly smaller overlap of selected genes (see Supporting Information Figure S4). Many genes were selected by only one of the platforms, indicating that the parametric framework does not account for other substantial differences that exist between platforms. Such conflicts suggest the inclusion of more false positives than expected for the test.

**Figure 4:**
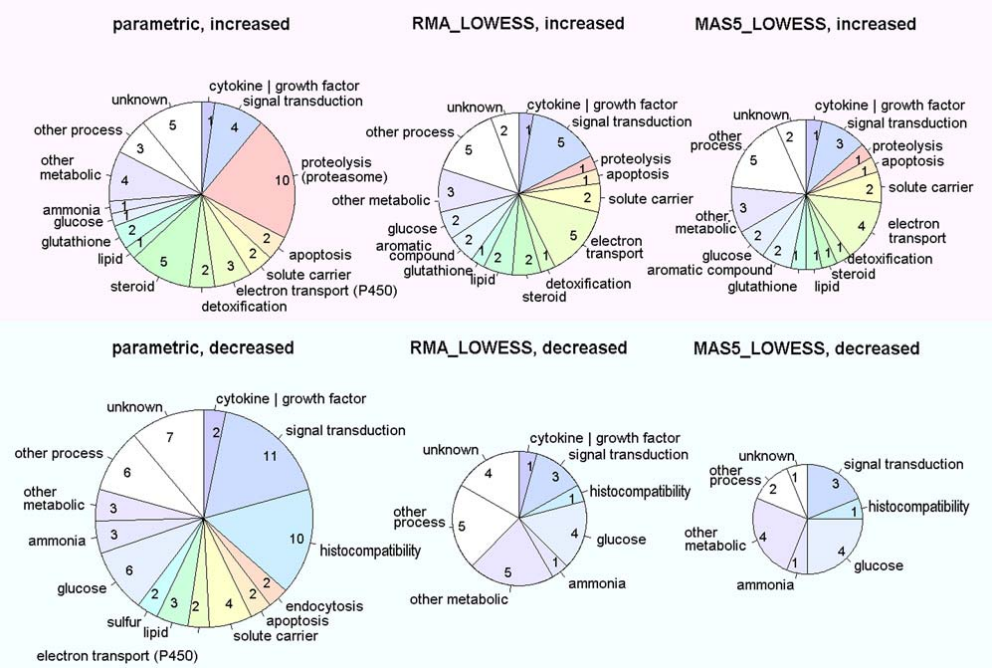
Functions of cross-selected genes in the toxicology study estimated from gene titles and biological processes of gene annotations provided by the chip manufacturer.

Trends in selected gene contents were also observed using the parametric framework. In estimating the physiological condition of the sample, the simultaneous selection of a group of genes indicative of a biological event is a more reliable indicator of that event than the selection of a single pertinent gene. In the present case, the parametric framework reveals an increase in genes related to proteolysis by proteasomes and metabolism of steroids, and a reduction in genes related to antigen presentation via MHC class II. These genes constituted large parts of the gene list (Figure 4, lower rows). The inclusion of many genes related to a specific function in the selected gene group results in a bias or tendency within the group that is different from that of the population. Such bias can be resolved by applying some of the key words included in the gene annotations for the chips. Table 1 shows the appearance ratio and *p* values for genes that include a key word for steroid metabolism, proteasome, or major histocompatible complex. Significant biases were only resolved by the parametric method. Additionally, out of 100 key words randomly selected from selected genes, 13 were judged to be significant by the parametric framework, whereas only 4 and 1 words of this group of 13 were determined to be significant by the RMA_LOWESS and MAS5_LOWESS frameworks, respectively. The superior sensitivity of the parametric framework for resolving such bias cannot be achieved simply by increasing the number of selected genes through adjustment of the threshold.

**Table 1:** List of ratios and *p* values of selected genes marked with a key word.

Increase	Parametric		RMA_LOWESS		MAS_LOWESS	
	Ratio	*p*	Ratio	*p*	Ratio	*p*
Cholesterol	4/47	2.7E-05	0/38	1	0/30	1
Steroid	6/47	9.6E-05	2/38	0.1	3/30	0.01
Macropain	9/47	2.1E-12	0/38	1	0/30	1
Proteasome	9/47	4.5E-12	0/38	1	0/30	1
Decrease	Ratio	*p*	Ratio	*p*	Ratio	*p*
Antigen	7/62	8.9E-08	0/25	1	0/17	1
Extracellular space	7/62	8.6E-05	1/25	0.2	2/17	0.03
Histocompatibility	2/62	8.4E-04	0/25	1	0/17	1
MHC & RT1	5/62	1.7E-06	0/25	1	0/17	1

## Discussion

The parametric framework appears to provide superior reproducibility with greater testing power and a lower false-positive rate compared to existing frameworks. In the present study, although the purpose and subjects of measurement were identical, the analytical results were not coincident between platforms. The degree of coincidence and conflict appears to be largely dependent on the framework employed for analysis of the acquired data. Many of the discrepancies in the information obtained from the two platforms considered here can be attributed to differences in the fundamental philosophies of the frameworks that have conventionally been applied to the respective platforms, and not to inherent differences in the capacities of the chip platforms. This is evidenced by the greater coincidence achieved between data acquired using different platforms when analyzed using the parametric framework.

Each of the frameworks normalizes and compares data using a set of hypotheses and assumptions that form the fundamental basis of the framework [1], [2]. In the parametric framework, chip data are normalized using a distribution model as the standard. Here the term “parametric” means a strict description with the least number of parameters and the least number of assumptions. On the other hand, a standard is sought among the data sets in the other frameworks. For example, the standard in other frameworks may be determined for a pair of data in LOWESS [16] and shift-log [18], and from the means of data quantiles in RMA. Consequently, the normalization of a data set in existing frameworks is affected by all of the data sets being processed at the same time. This dependency on other data sets can be expected to adversely affect the uniformity of the analysis, which becomes apparent when comparing information among different studies.

Another fundamental difference is associated with the testability of the fundamentals of the framework. LOWESS and quantile methods including RMA inevitably fulfill the assumptions regarding the assumed nature of the data, that is, that data take the form of stable logarithmic ratios (LOWESS) or identical data distributions (quantile method). MAS5 contains numerous conditional judgments that are not based on factual knowledge. The premises of these frameworks therefore preclude effective evaluation of the model assumptions. The parametric framework, on the other hand, employs a strict model and normalization cannot be completed without coincidence between the model and chip data. Any test of validity therefore relates to the reliability of the obtained information.

The normalization process, which tests data distribution against a model (Figure 1), is useful for identifying the likely range of data. As with other measurement systems, microarrays inevitably contain noise. With repeated measurements such as those shown in Figure 3, the noise level can be reduced by taking the means of repeats. However, the noise contained in each measurement may still affect analyses, particularly when small numbers of repeats are available. Even in such cases, the parametric framework allows the data range that is likely to be affected by additive noise and saturation to be clearly defined (Figure 1). The usefulness of this method for data classification is clearly shown in Figure 2, and is expected to increase the reliability of analyses by reducing the false-positive rate.

The parsimonious character of the proposed parametric framework assists in maintaining objectivity in analyses. As the summarization of data with perfect objectivity in order to obtain abstract representations of measurements such as shown in Figure 4 is quite difficult, analysts tend to depend on personal criteria to judge the importance of each piece of information. However, by the proposed approach, the false-positives appearing in the abstracted data can be controlled, allowing significant increases in cell function apparent in the microarray data to be tested by reference to the binominal distribution model (Table 1). If the function is significant, many of the related key words will become significant by this test. On the other hand, if only a limited part of the key words tested show significance, the estimation of cell function should be reconsidered by altering the category of the function or by focusing on the speciality of the function. It should be noted that this test can cause multiple comparison problems, as false-positives will occur in accordance to the threshold value. Furthermore, some positive key words will not directly represent the probability of functional change, since many key words are not independently used in annotations, and may be correlated. However, such factors will not disturb the testing of each word or phrase. The present tests clearly show that the proposed parametric framework has superior sensitivity compared to the other frameworks evaluated in this study, corresponding to lower levels of false selection of genes.

The proposed parametric framework thus achieves superior universality of data and allows for the evaluation of data reliability, thereby providing a means of integrating knowledge obtained from many different laboratories and chip platforms.

## Materials and Methods

### Test animals and microarrays used in the toxicology study

Male Fischer 344 rats (SPF, 5 weeks of age) were administered with 300 mg/kg/day of Safrol for up to 28 days at the Mitsubishi Chemical Safety Institute [11], [12]. RNA samples were isolated from the liver of each test animal. Identical RNA samples were investigated using GeneChip (Rat Genome 230 2.0 array; Affymetrix) and a NEDO-ToxArray III ink-jet printed chip (6709 genes [10]). These microarrays share an overlap of 4433 genes.

### Data obtained from the Gene Expression Omnibus

Data sets employed in a platform comparison study [13] were obtained from the Gene Expression Omnibus [19]. In the comparison study, two batches of RNA (mouse cortex samples and retina samples) were hybridized to 9 platforms: ABI, Amersham, and Mergen (single-color slides); Agilent, Cepko, MGH, MWG, and Operon Compugen (two-color slides); and Affymetrix (GeneChip). The present parametric normalization scheme was applied to the raw signal intensity data for the slide glass-type chips and the file data (CEL) for GeneChip assays. Amersham data was not tested due to the non-availability of raw data. Mergen and Cepko data were not analyzed due to the lack of information to find correspondences to the other platforms.

### Normalization

Parametric normalization was performed using SuperNORM (Skylight Biotech, Akita). Other normalizations were performed using *R* version 2.4 [20] with the implemented *affy* library [21] as follows. The scanner-estimated background was subtracted from the Cy3 and Cy5 data for each ToxArray chip and the printed chips in the platform comparison study, and the logarithmic ratios were stabilized using the LOWESS function [16] in *R*. The GeneChip data were normalized and summarized using the MAS5 function or the RMA [15] function of *affy* [21]. The data obtained using Agilent chips were processed by parametric scanning [17] in order to eliminate data affected by hybridization unevenness. This step could not be performed for the other chips due to the lack of necessary information, such as the position of each spot on the chip. For the remaining data sets from the platform comparison study, donated normalized values were used.

### Estimation of differences in expression levels

The expressional difference for each gene (*E_g_*) between samples *T* and *C* are determined from the means of measurements using the logarithmic forms of the normalized data for the each chip, as follows.
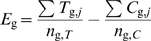
Here, *n*_g,*T*_ and *n*_g,*C*_ are the numbers of available data for samples *T* and *C*, and *i* denotes the *i*th chip. Four samples (*n* = 4) were assigned for treatment and control in the toxicology study, and *n* = 5 samples were taken for pooled cortex and retina assay in the platform comparison data. The logratio values are estimated by unifying the bases of the logarithms to base 2. The parametric framework data were further adjusted with respect to *E*_g_ by multiplication by the mean σ values of measurements [9]. The logratio values are compared between array platforms in Figure 2.

### Comparisons of selected genes

Gene selection was performed for the toxicology and platform comparison projects using different criteria, although both criteria are based on the expressional differences and noise levels in measurements. The differences among frameworks are illustrated by comparing the genes selected by slide-glass-type chips with those selected by GeneChip assays. The areas in each diagram denote the proportion of corresponding genes. The criteria employed for the toxicology study are as follows. The priority of selection assigned to the sensitivity in detecting expressional changes, since the magnitude of differences and the number of affected genes are expected to be limited. To maximize the sensitivity, the thresholds were set in consideration of the noise level of measurement. The noise level was determined from the standard deviation of the fluctuations in the normalized logarithmic data. The standard deviation was estimated using the mean of sample variances (*s*_g_^2^), which was measured for each gene as follows.
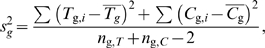
where *T̅* and *C̅* represent the mean of data *T* and *C*, respectively. The threshold level (*L*) was then determined using the expression
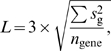
where *n*_gene_ is the number of genes. Genes with |*E_g_*| values larger than *L* were selected. Assuming that the effects of noise on the logarithmic data are normally distributed, the false-positive rate of selection is estimated to be 0.27% in the subject genes. In the parametric framework, only data in the valid range were used for selection. In the GeneChip data, differences were estimated at each PM data level. In MAS5, only “Present” data were used. Genes without at least 3 cells making successful measurements within the model-associated signal range in both control and treatment were excluded.

The criteria used for selected data from the platform comparison studies were as follows. As differences originate from the differences between the two organs assayed, excessive numbers of genes would be selected by the previous criterion. Therefore, selection was performed by applying a more stringent criterion, where only those genes with differences in expression levels having a *p* value of less than 0.01 and a two-fold variation being selected. In the estimation of *p* values, a paired *t* test was applied for the two-color printed chips (pairs were formed in a chip-wise manner giving 5 pairs of data per gene), an unpaired *t* test was applied for single-color chips (5 data per gene), and a variance analysis was applied for GeneChip data by gene-wise pairing in accordance with PM data (20 pairs of PM data, 5 chips per gene). Forming the pairs in a chip-wise manner has the advantage of reducing the number of defects due to uneven hybridization (data not shown).

### Test of bias in annotation key words among selected genes

Augmentation of the specific functions of a cell results in an increase in the transcripts of genes related to those functions. If “positive genes” are sought by microarray assay, many such stimulated genes will be selected. Consequently, the selected group of genes will exhibit trends in the functions of genes, resulting in a bias in the population of chip contents. Such bias can be detected in the key words appearing in the annotations for selected genes. For a certain key word, the appearance rate, rate_key_, can be defined as the proportion of genes for which that key word appears in the annotations. For a randomly selected group of genes from the population, the number of “key”-annotated genes in the group will follow a binominal distribution. The probability that the number of key genes is equal or larger than *n* in *m* selected genes can be expressed by a cumulative distribution function, as given by
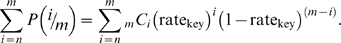



When the probability is less than a threshold value, the appearance rate of “key”-annotated genes in the group is deemed to be significantly high. In the present study, this procedure was applied to 100 randomly selected words or phrases, and to words that represent issues apparent in the diagrams shown in Figure 4. Evidence for all the words included in the latter test are listed in Table 1. Random words were chosen from the annotations of 921 genes selected in at least one of the methods or platforms. The threshold of the test was 0.001, corresponding to one false-positive in every 1000 key words. For example, the word “steroid” appeared in 70 genes in 4433 overlapping contents of ToxArray and GeneChip, yielding ratio_steroid_ value of 0.0158. The probability that 6 or more genes are included in 47 randomly selected genes is therefore 9.6E-05. This value is far less than the threshold, suggesting that the proportion of steroid-related genes is significantly high.

## Supporting Information

Figure S1 Data distribution of NEDO ToxArray III chip and GeneChip
(0.08 MB DOC)

Figure S2 Pseudo Images of NEDO ToxArray III and GeneChip
(1.31 MB DOC)

Figure S3 Effect of 2-Acetylaminofluorene (2-AAF).
(0.06 MB DOC)

Figure S4 Reproducibility Between Means among Repeated Measurements.
(0.05 MB DOC)

Supporting Information S1 Data simulation in the R. Affection of noise to lognormal distribution.
(0.02 MB DOC)
