# Supplementary figures and images for: Coincidence between Transcriptome Analyses on Different Microarray Platforms Using a Parametric Framework
Source: PLoS One. 2008 Oct 29;3(10):e3555. doi: 10.1371/journal.pone.0003555 (PMC2570215; doi:10.1371/journal.pone.0003555)

NEDO ToxArray III


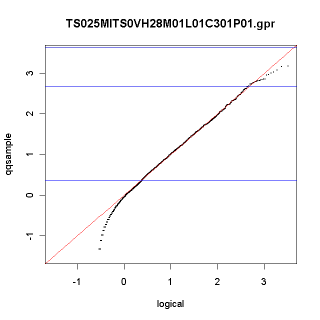


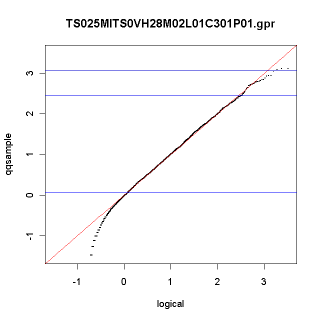

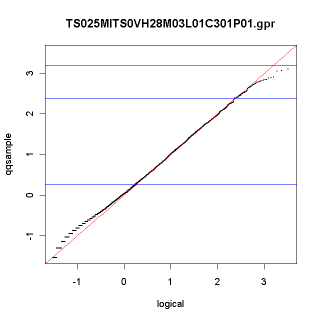

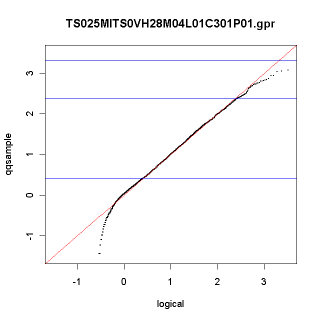


GeneChip


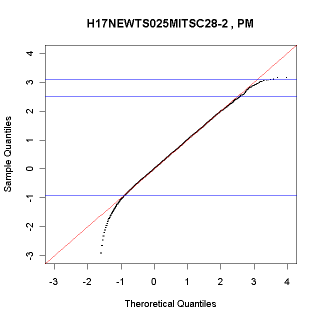

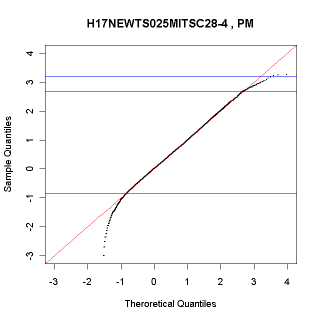


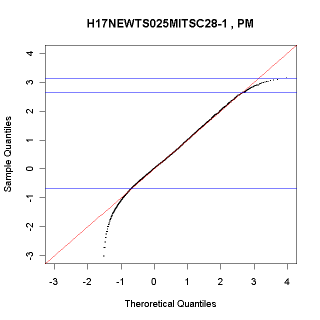

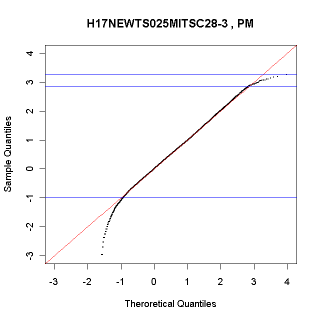

Supplement: Figure S1 — Data distribution of NEDO ToxArray III chip and GeneChip (0.08 MB DOC) [file pone.0003555.s001.doc]

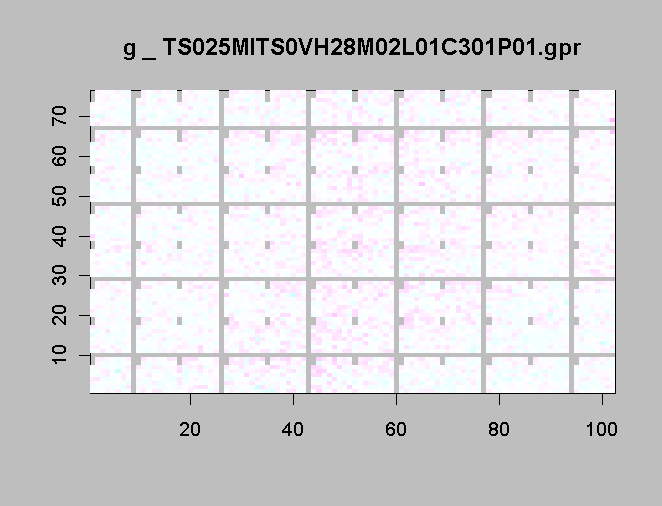
NEDO ToxArray III


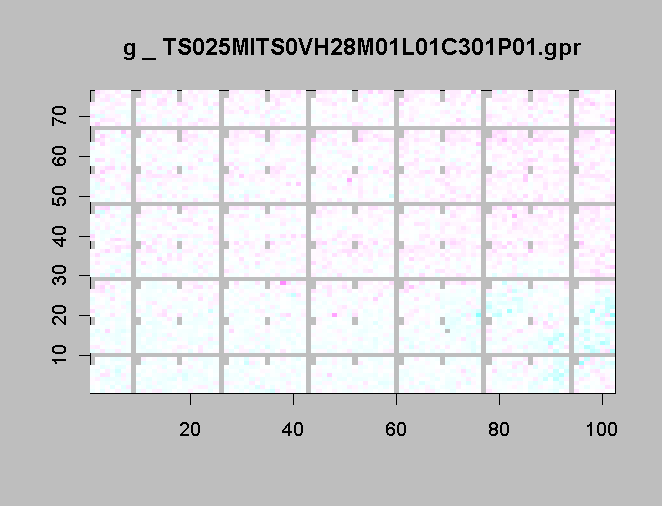


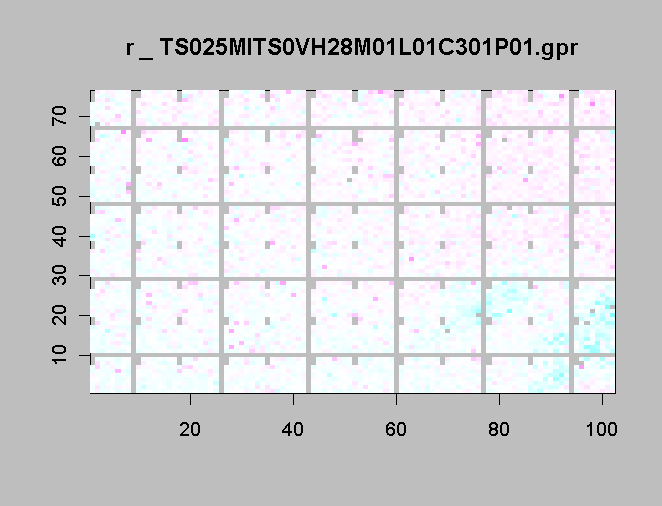

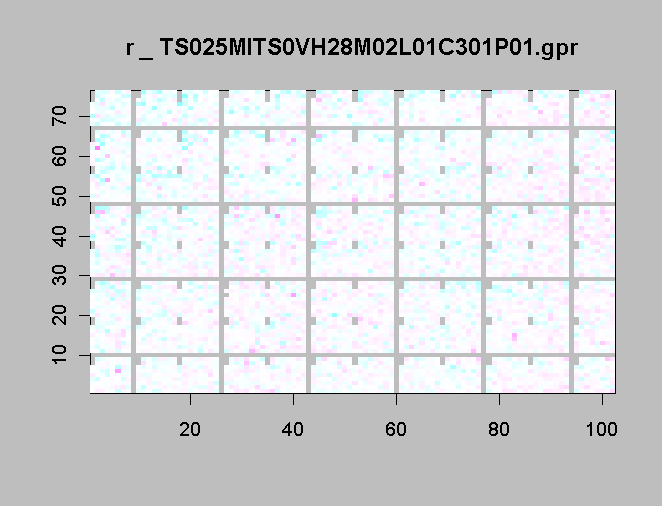




GeneChip


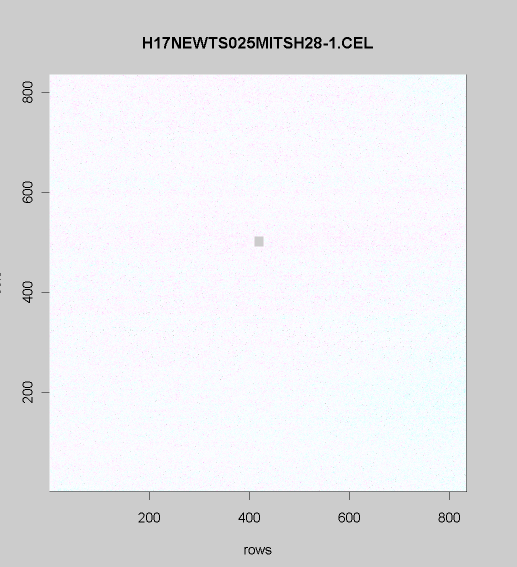

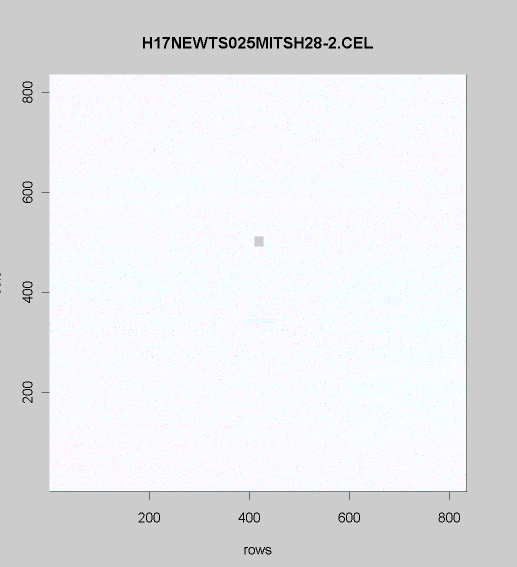

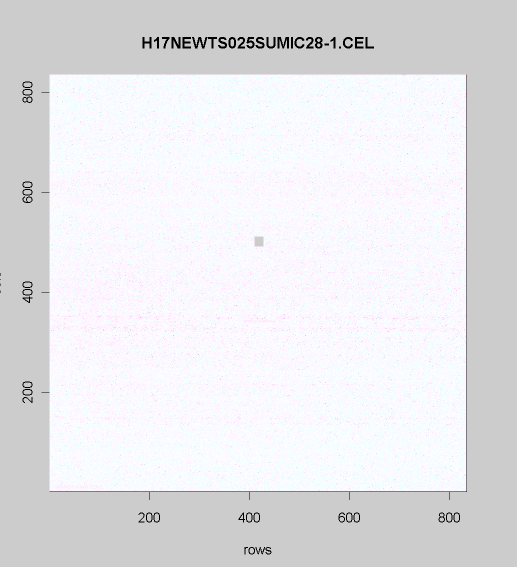

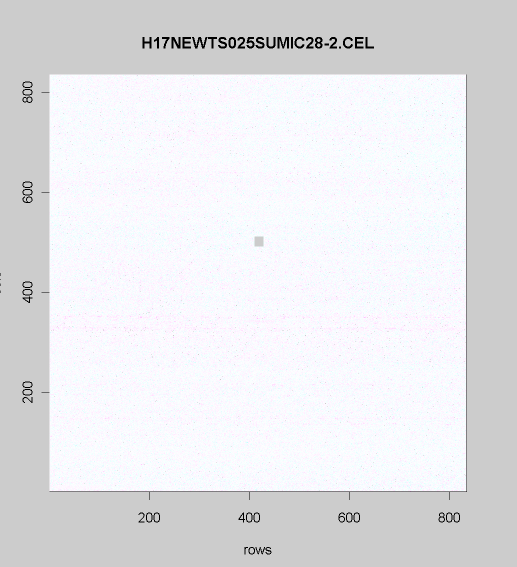


Supplement: Figure S2 — Pseudo Images of NEDO ToxArray III and GeneChip (1.31 MB DOC) [file pone.0003555.s002.doc]
