# Supplementary material for: Coincidence between Transcriptome Analyses on Different Microarray Platforms Using a Parametric Framework
Source: PLoS One. 2008 Oct 29;3(10):e3555. doi: 10.1371/journal.pone.0003555 (PMC2570215; doi:10.1371/journal.pone.0003555)
Supplement: Figure S3 — Effect of 2-Acetylaminofluorene (2-AAF). (0.06 MB DOC) [file pone.0003555.s003.doc]

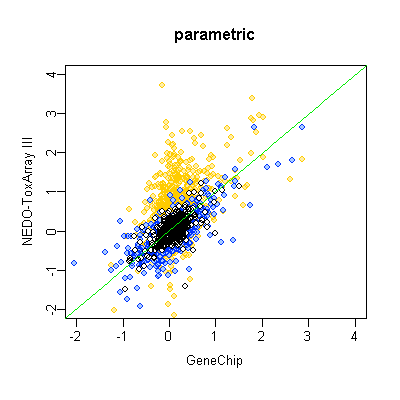

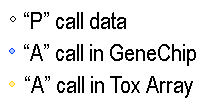

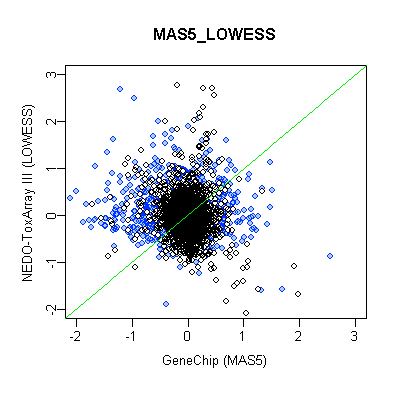

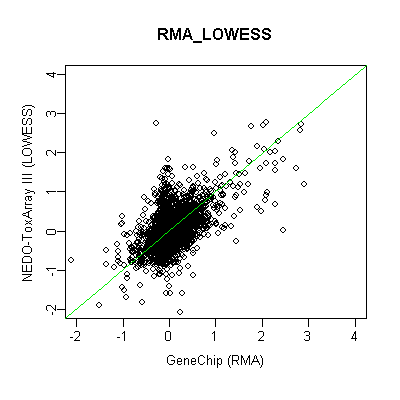


Figure S3

Effect of 2-Acetylaminofluorene (2-AAF). Differences found at 14th day of 2-AAF treatment. Data obtained from *one* GeneChip and *one* ToxArray are compared. It should be noted that the magnitude of the differences are smaller than those found in the case of 25th day of Safrol treatment (see Fig. 2 in the text).The colored spots present "A" called data: "Absent" in MAS5 or "Affected by noise" in the parametric framework.
