# Supplementary material for: Coincidence between Transcriptome Analyses on Different Microarray Platforms Using a Parametric Framework
Source: PLoS One. 2008 Oct 29;3(10):e3555. doi: 10.1371/journal.pone.0003555 (PMC2570215; doi:10.1371/journal.pone.0003555)
Supplement: Figure S4 — Reproducibility Between Means among Repeated Measurements. (0.05 MB DOC) [file pone.0003555.s004.doc]

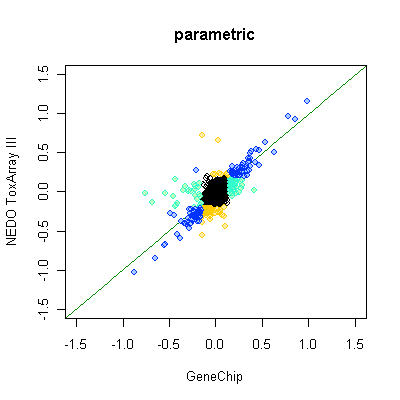


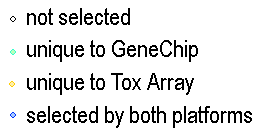


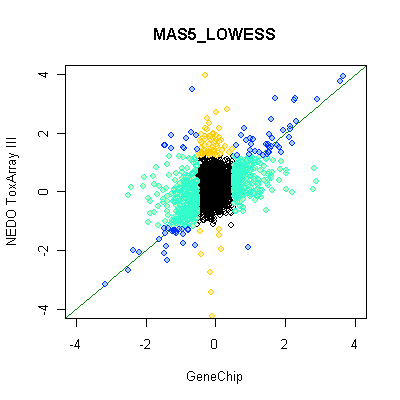

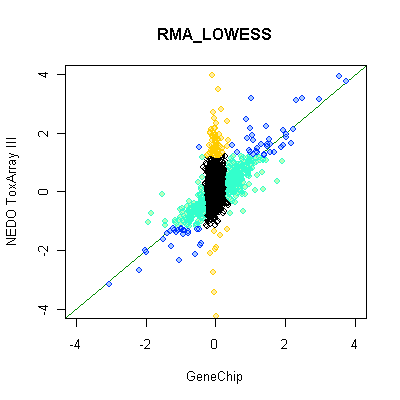


Figure S4

Reproducibility Between Means among Repeated Measurements. Differences found at 25th day of Safrol treatment (the same data used in Fig. 3 in the text are presented).

Means of data obtained from GeneChips (n=4 for both control and treatment) and ToxArray chips are compared. Colored plots present the selected genes.
