# Supplementary material for: Coincidence between Transcriptome Analyses on Different Microarray Platforms Using a Parametric Framework
Source: PLoS One. 2008 Oct 29;3(10):e3555. doi: 10.1371/journal.pone.0003555 (PMC2570215; doi:10.1371/journal.pone.0003555)
Supplement: Supporting Information S1 — Data simulation in the R. Affection of noise to lognormal distribution. (0.02 MB DOC) [file pone.0003555.s005.doc]

data simulation (affection of noise to lognormal distribution) in *R*

# the theoretical values of lognormal distribution

lognormalsignal <- 10^qnorm(ppoints(10000), sd=1, mean=0)

#adding additive noise to the lognormal series

lognormalsignal_noise <- lognormalsignal+rnorm(10000, sd=0.01, mean=0)

#comparing the theoretical value and the noise-containing series

plot(log10(lognormalsignal) , sort(log10(lognormalsignal_noise), na.last=F ), ylim=c(-4,4), col=rainbow(10)[1])

#repeating the above with different noise levels

lognormalsignal_noise <- lognormalsignal+rnorm(10000, sd=0.01*5, mean=0)

par(new=T)

plot(log10(lognormalsignal) , sort(log10(lognormalsignal_noise), na.last=F ), ylim=c(-4,4), col=rainbow(10)[2])

lognormalsignal_noise <- lognormalsignal+rnorm(10000, sd=0.01*5*5, mean=0)

par(new=T)

plot(log10(lognormalsignal) , sort(log10(lognormalsignal_noise), na.last=F ), ylim=c(-4,4), col=rainbow(10)[3])

lognormalsignal_noise <- lognormalsignal+rnorm(10000, sd=0.01*5*5*5, mean=0)

par(new=T)

plot(log10(lognormalsignal) , sort(log10(lognormalsignal_noise), na.last=F ), ylim=c(-4,4), col=rainbow(10)[7])

par(new=T)

plot(log10(lognormalsignal) , log10(lognormalsignal), ylim=c(-4,4), col="black")
